# Assessing medical impoverishment and associated factors in health care in Ethiopia

**DOI:** 10.1186/s12914-020-00227-x

**Published:** 2020-03-30

**Authors:** Amarech G. Obse, John E. Ataguba

**Affiliations:** Health Economics Unit, School of Public Health and Family Medicine, Health Sciences Faculty, University of Cape Town, Anzio Road, Observatory, Cape Town, 7925 South Africa

**Keywords:** Financial protection, Impoverishment, out-of-pocket health spending, Ethiopia

## Abstract

**Background:**

About 5% of the global population, predominantly in low- and middle-income countries, is forced into poverty because of out-of-pocket (OOP) health spending. In most countries in sub-Saharan Africa, the share of OOP health spending in current health expenditure exceeds 35%, increasing the likelihood of impoverishment. In Ethiopia, OOP payments remained high at 37% of current health expenditure in 2016. This study assesses the impoverishment resulting from OOP health spending in Ethiopia and the associated factors.

**Methods:**

This paper uses data from the Ethiopian Household Consumption Expenditure Survey (HCES) 2010/11. The HCES covered 10,368 rural and 17,664 urban households. OOP health spending includes spending on various outpatient and inpatient services. Impoverishing impact of OOP health spending was estimated by comparing poverty estimates before and after OOP health spending. A probit model was used to assess factors that are associated with impoverishment.

**Results:**

Using the Ethiopian national poverty line of Birr 3781 per person per year (equivalent to US$2.10 per day), OOP health spending pushed about 1.19% of the population (i.e. over 957,169 individuals) into poverty. At the regional level, impoverishment ranged between 2.35% in Harari and 0.35% in Addis Ababa. Living in rural areas (highland, moderate, or lowland) increased the likelihood of impoverishment compared to residing in an urban area. Households headed by males and adults with formal education are less likely to be impoverished by OOP health spending, compared to their counterparts.

**Conclusion:**

In Ethiopia, OOP health spending impoverishes a significant number of the population. Although the country had piloted and initiated many reforms, e.g. the fee waiver system and community-based health insurance, a significant proportion of the population still lacks financial protection. The estimates of impoverishment from out-of-pocket payments reported in this paper do not consider individuals that are already poor before paying out-of-pocket for health services. It is important to note that this population may either face deepening poverty or forgo healthcare services if a need arises. More is therefore required to provide financial protection to achieve universal health coverage in Ethiopia, where the informal sector is relatively large.

## Background

Understanding the nexus between poverty and (ill) health is essential for development, especially within the Sustainable Development Goals (SDGs) [1–3]. Poverty levels remain high in sub-Saharan Africa (SSA), accounting for 42% of global extreme poverty headcount in 2013 [4]. While poverty levels are high, another possible contributor to poverty, out-of-pocket (OOP) health spending, was estimated at 35% of current health expenditure in SSA in 2016 [5]. Generally, OOP health spending that is greater than 20% of current health expenditures reduces the likelihood of financial protection in a country’s health system [6]. In SSA, OOP health spending exceeds 20% of current health expenditure in more than 70% of the countries [5], predisposing a substantial proportion of the population to impoverishment [7]. In 2010, globally, over 95 million people were impoverished by OOP health spending, falling below the $1.90 international poverty line [3]. In fact, the World Health Organization (WHO) estimates that each year, OOP health spending impoverishes up to 5% of the global population [8], but standard poverty measures do not account for this impoverishing effect of OOP health spending [7].

Ethiopia, like most countries in SSA, finances health services, among other mechanisms, through OOP health spending which has increased from 33.7% (in 2010/2011) [9] to 37.0% (in 2016) of the country’s current health expenditure [5]. Private health centres and clinics – including all types of clinics – account for 65.7% of all health centres in Ethiopia [10]. User fees still exist at public facilities, and private facilities charge OOP for health services. The significant number of private health centres implies that a substantial number of people would be seeking care from private facilities, incurring significant OOP health spending. Public health facilities in Ethiopia implement a fee-waiver system aimed at exempting the poorest from fees.

Since 1999/2000, Ethiopia has contemplated different health financing reforms that aimed at both the supply-side (the first generation reforms – strengthening the health system) and demand-side (the second generation reforms – introducing health insurance schemes) to improve the attainment of the health system goals [11]. Increasing health system resources by improving efficiency and raising more revenue was one of the major policy objectives in the first-generation reforms. In fact, most of the supply-side reforms contribute to this objective. Some of these reforms were revenue raising and retention by healthcare facilities, improving the fee-waiver system, establishing a private wing in public facilities, and outsourcing of non-clinical services. As a result of these reforms, there was a significant increase in health sector resources. For instance, per capita health expenditure increased from $5.60 (1999/00) to $7.14 (2004/05) and $20.77 (2010/11) [9]. However, the impact of the first generation reforms in reducing OOP health spending was minimal. OOP health spending as a share of current health expenditure dropped only by two percentage points between 1999/00 (36%) and 2010/11 (34%)^1^ as reported in the Ethiopian National Health Accounts [9, 12]. The existence of high OOP health spending remained a critical issue in policy documents, and among the reforms proposed were establishing health insurance schemes; which are considered to be the second generation reforms [11, 13]. Social health insurance (SHI) was established under the proclamation number 690/2010 of the Federal Democratic Republic of Ethiopia (FDRE), while community-based health insurance (CBHI) has been piloted since mid-2011 in different regions of the country [14–16]. The health insurance system is still in the early development stages, and more is needed to contribute to a significant reduction in OOP health spending below 20% of current health expenditure to guarantee financial protection.

In Ethiopia, an assessment of the impoverishing impact of OOP health spending in 2004 report that less than 1% of the population was pushed into poverty [17]. However, levels of impoverishment vary by household characteristics. For instance, a higher rate of impoverishment (5.8% points) is reported for households of persons with depression compared to households without persons with depression [18]. These findings suggest the need for further studies on the factors associated with impoverishment and an update in the assessment of poverty levels related to OOP health spending using recent datasets. Such a study can also show the impact of health financing reforms that were introduced since 1999/00. This paper, therefore, assesses the effect of OOP health spending on monetary poverty in Ethiopia to evaluate the country’s progress in achieving universal financial protection. It also examines the factors that are associated with impoverishing OOP health spending.

## Methods

### Data

This paper uses the Ethiopian Household Consumption Expenditure Survey (HCES) 2010/11, the latest data available when this study was conducted. The HCES is a nationally representative survey conducted by the Ethiopian Central Statistical Agency using the 2007 Population and Housing Census sampling frame. The HCES uses a two-stage stratified sampling design. The first stage involved the selection of 864 rural and 1104 urban Enumeration Areas (EAs) from the 11 regions of the country except for some areas in two regions with a nomadic population. A total of 10,368 rural and 17,664 urban households are selected from the sampled EAs [19]. The HCES contains data on household expenditure on goods and services, including OOP payments for various health services (including consultation, diagnosis, tests, and medicine). Household expenditure is used as a proxy for income because income is not reliable in many African settings. Per capita household expenditure, computed by dividing each household’s total expenditure by their total household size, was used for the assessment of impoverishment.

### Analysis of the impoverishing impact of out-of-pocket health spending

Impoverishing impact of OOP health spending is estimated at the individual level by comparing poverty levels before (pre-payment) and after (post-payment) OOP health spending. Three measures of poverty are estimated: poverty headcount ratio, poverty gap, and the normalised poverty gap. These indices are estimated using both prepayment and post-payment per capita expenditure. Prepayment expenditure represents the household budget before paying OOP for health services, while post-payment expenditure is prepayment expenditure net of OOP health spending. The poverty headcount index measures the proportion of individuals with per capita consumption expenditure (pre- or post-payment) below the poverty line [20].

As outlined elsewhere [21], if ${P}_i^{pre}$ is the prepayment poverty indicator, such that ${P}_i^{pre}=1\left({y}_i< PL\right)$, it means that an individual *i* is poor if *y*_*i*_ < *PL* . Here, *y*_*i*_ is the individual’s per capita prepayment expenditure and *PL* is the poverty line.

The pre-payment poverty headcount ratio (${H}_{pov}^{pre}$) is given as:

$$ {H}_{pov}^{pre}=\frac{1}{N}\sum \limits_{i=1}^N{P}_i^{pre}={\mu}_{P^{pre}} $$

where *N* is to total sample size. Further, if we define ${g}_i^{pre}$ as the deficit from the poverty line such that:

$$ {g}_i^{pre}={P}_i^{pre}\left( PL-{y}_i\right) $$

Then the prepayment poverty gap measure (${G}_{pov}^{pre}$) that aggregates the deficit of household per capita expenditures from the poverty line is given as:

$$ {G}_{pov}^{pre}=\frac{1}{N}\sum \limits_{i=1}^N{g}_i^{pre}={\mu}_{g^{pre}} $$

The prepayment poverty gap normalised by the poverty line (${NG}_{pov}^{pre}$) is given as:

$$ {NG}_{pov}^{pre}={G}_{pov}^{pre}/ PL $$

The normalised poverty gap is useful to compare poverty levels between countries with different currencies and poverty lines [22].

The post-payment poverty indices $\left({H}_{pov}^{post},{G}_{pov}^{post},{NG}_{pov}^{post}\right)$ are obtained analogously by replacing the prepayment expenditure by post-payment expenditure.

The difference between the post-payment and prepayment poverty indices provides the poverty impact of OOP payments. For example, the impact of OOP health expenditures on the poverty headcount is $$ \left({H}_{pov}^{post}-{H}_{pov}^{pre}\right) $$. Two poverty lines were used in this paper—the US$1.90/day international poverty line at the 2011 purchasing power parity (PPP) [23] and the national poverty line (Birr 3781 per person per year [24]); which is equivalent to US$2.10/day in 2011 prices.

The probit regression was used to assess the factors associated with impoverishment headcount based on the national poverty line. The dependent variable in the probit regression is a binary indicator that takes on the value of one for impoverishment from OOP health spending and zero, otherwise. The explanatory variables in the model included household-level factors selected based on literature and availability in the dataset [25]. Factors related to household head are age, gender, education, marital status, and whether the household head engaged in any productive work. Other household-level covariates include household size, availability of under-five children, availability of adults aged at least 65 years, and the ecology of residential areas (highland, moderate, lowland, urban).

The “Pen’s parade of dwarfs and a few giants” was used to depict the impoverishing effect of OOP health spending [22]. Here, prepayment consumption expenditure is graphed against post-payment consumption expenditure, with individuals ordered according to prepayment consumption expenditure. Stata 15 [26] was used to analyse the data.

## Results

### Descriptive statistics

Over 60% of the households are in urban areas, with an average of five members per household (Table 1). The average age of the household heads was 42 years. About two-thirds of the households were male-headed. A large proportion of household heads (43.10%) had no formal education. Only about 31.19 and 19.31% attained primary and secondary education levels, respectively. The average annual per capita household consumption expenditure and OOP health spending were Birr 30,088 (US$ 1751.34) and Birr 431 (US$ 25.09), respectively.

**Table 1:** Descriptive characteristics of households

	Weighted percentage/ average
**Sex of the household head**	
Male	75.09
Female	24.91
**Education level of the household head**	
Primary	22.76
Secondary	4.67
Tertiary	2.14
Informal education/Illiterate	71.24
**Marital status of the household head**	
Married	73.97
Never married	6.31
Other	19.72
**Employment status of the household head**	
Employed	91.17
Unpaid work/unemployed	8.83
**Place of residence of the household**	
Urban	21.45
Rural	78.55
Age of household head	42.00
Household size	5.00
Out-of-pocket payment per capita (in Birr)	431.00
Household total consumption expenditure per capita (in Birr)	30,088.00

1 USD equals 17.18 Ethiopian Birr in 2011

### Estimates of impoverishment

The national poverty headcount ratio increased by 1.19% points due to OOP health spending, using the Ethiopian national poverty line of US$2.10/day (Table 2). At the same poverty line, the normalised poverty gap rose by 0.60% points, representing a 4.51% point relative increase. The impoverishing effect of OOP health spending was very similar, albeit lower, using the US$1.90/day international poverty line. For example, poverty headcount increased by 1.18% points, while the average normalised poverty gap increased by less than 0.01% points.

**Table 2:** The impoverishing effect of OOP health spending in Ethiopia, 2010/11

	Poverty headcount	Poverty gap	Normalised gap	Normalised mean positive gap
**Ethiopian Poverty line (Birr 3781 (US$2.10) per person per year)**				
Pre-payment	45.26%	506.18	13.39%	29.58%
Post-payment	46.45%	529.00	13.99%	30.12%
Absolute difference	1.19%	22.82	0.60%	0.54%
Relative difference	2.62%	0.05	4.51%	1.84%
**International poverty line (US$ 1.90 per person per day)**				
Pre-payment	37.24%	358.79	10.49%	28.16%
Post-payment	38.41%	377.08	10.49%	28.69%
Absolute difference	1.18%	18.29	< 0.01%	0.53%
Relative difference	3.16%	0.05	< 0.01%	1.88%

The analysis of medical impoverishment by regions, using the national poverty line, shows that Harrari, SNNP, and Somali regions have very high rates of impoverishment due to OOP health spending with 2.35, 1.66, and 1.36 percentage point increases in the fraction of people pushed into poverty, respectively. Impoverishment in Addis Ababa was the least, with only about 0.35% of the population pushed into poverty by OOP health spending. However, the absolute depth of the rise in poverty was higher for SNNP, Oromia, and Afar where the normalised poverty gaps increased by 0.71, 0.68, 0.58 percentage points, respectively. In terms of residential areas, the rise in poverty levels and absolute intensity of poverty was higher in rural than in urban areas. The poverty headcount ratio rose by 1.31 (rural) and 0.60 (urban) percentage points due to OOP health payments while the increases in the normalised poverty gaps were 0.69 and 0.21 percentage points, respectively. Although the rise in headcount ratio was higher for female-headed than male-headed households, the rise in absolute normalised poverty gaps was larger for male-headed households (Table 3).

**Table 3:** The impoverishing effect of OOP health spending by region, residential area, and gender in Ethiopia, 2010/11

	Poverty headcount			Normalised gap		
	Pre	Post	Absolute difference	Pre	Post	Absolute difference
**By region**						
Tigray	37.30%	37.69%	0.39%	9.79%	10.20%	0.41%
Afar	40.93%	41.51%	0.58%	10.40%	10.98%	0.58%
Amhara	48.14%	49.39%	1.25%	14.47%	14.97%	0.50%
Oromia	43.48%	44.58%	1.10%	12.61%	13.29%	0.68%
Somali	43.74%	45.10%	1.36%	11.18%	11.56%	0.38%
Benishangul-Gumuz	42.33%	43.45%	1.12%	11.98%	12.54%	0.56%
SNNP	54.92%	56.59%	1.66%	17.26%	17.97%	0.71%
Gambella	37.46%	38.54%	1.08%	9.86%	10.25%	0.39%
Harari	12.31%	14.66%	2.35%	1.98%	2.20%	0.22%
Addis Ababa	11.49%	11.84%	0.35%	2.62%	2.87%	0.25%
Dire Dawa	24.85%	25.66%	0.81%	3.66%	3.83%	0.17%
**By residential area**						
Urban	14.99%	15.59%	0.60%	3.37%	3.58%	0.21%
Rural	51.59%	52.90%	1.31%	15.48%	16.17%	0.69%
**By gender**						
Male	46.54%	47.70%	1.16%	13.78%	14.40%	0.62%
Female	38.70%	40.01%	1.31%	11.36%	11.89%	0.53%

The results of the probit regression model show that living in a rural area is the main predictor of impoverishment from OOP health spending. The probability of impoverishment increases by 30.6, 34.1, and 33.6 percentage points for people in highland, moderate, and lowland rural areas, respectively, as compared to those in urban areas. Households with at least one under 5-year-old child (12.9% points) and larger households (4.9% points) also have a significantly higher probability of experiencing impoverishing OOP health spending compared to their counterparts. The probability of impoverishing OOP health spending reduces by 10.2% points in households headed by an individual with formal education compared to those headed by an individual with no formal education. Although male-headed households have a reduced likelihood of experiencing impoverishing OOP health payments, this is only marginally significant (Table 4).

**Table 4:** Factors associated with impoverishing OOP health spending in Ethiopia, 2010/11

	Coefficients (Standard errors)	Marginal effects (Standard errors)
Age of household head (in years)	< 0.001 (0.002)	< 0.001 (0.001)
Household size	0.124*** (0.019)	0.049*** (0.008)
Location: Rural highland (reference: urban area)	0.800*** (0.077)	0.306*** (0.026)
Location: Rural moderate (reference: urban area)	0.890*** (0.065)	0.341*** (0.026)
Location: Rural lowland (reference: urban area)	0.875*** (0.079)	0.336*** (0.027)
Male headed household (reference: female-headed household)	−0.133* (0.069)	− 0.053* (0.027)
Household head with formal education (reference: household head without formal education)	−0.269*** (0.063)	− 0.106*** (0.024)
Household head married (reference: household head not married)	0.018 (0.066)	0.007 (0.026)
Household head had productive work (reference: household head without any productive work)	−0.050 (0.071)	− 0.020 (0.028)
Household with at least one child aged under 5 years(reference: household without any child under 5 years)	0.329*** (0.056)	0.129*** (0.021)
Household with at least one adult aged over 65 years (reference: household without an individual aged over 65 years)	0.045 (0.079)	0.018 (0.031)
Constant	−1.557*** (0.118)	
*N*	15,958	

*, *** denote statistical significance at the 10 and 1% levels, respectively

**Fig. 1:**
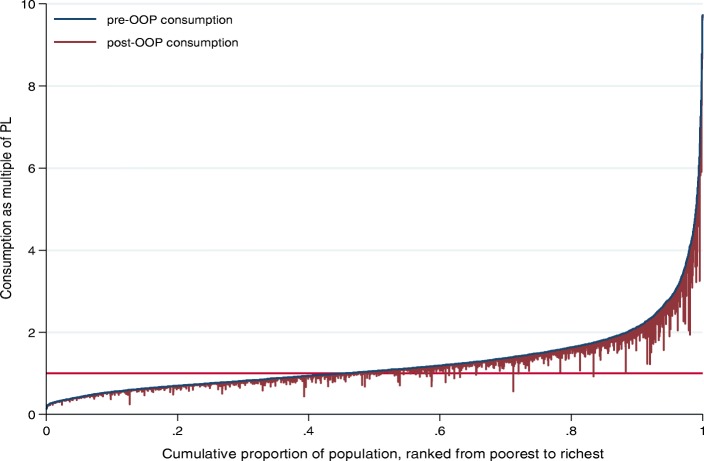
Effect of OOP health spending on the Pen’s parade of the household consumption, 2010/11. *Note*: This uses the national poverty line (US$2.10/day)

Figure 1 depicts the impoverishing effect of OOP health spending using the Pen’s parade. This figure shows that some non-poor individuals are dipped into poverty while many already poor individuals became further impoverished by OOP health spending in Ethiopia.

## Discussion

This study presented an analysis of the impoverishing effect of OOP health spending and factors associated with impoverishing OOP health spending in Ethiopia. In 2011, between 1.18 and 1.19% of the total population were forced into poverty in Ethiopia due to OOP health spending. When extrapolated at the national level based on population projection for 2011 [27], this represents over 957,100 individuals impoverished by OOP health spending. Also, the average poverty gap has deepened for those who were already below the poverty line before paying OOP for health services. Harari, SNNP, Somali, and Amhara regions had higher incidences of impoverishment due to OOP health spending, which is higher than the national average. However, SNNP, Oromia, and Afar had a more substantial rise in the depth of poverty, part of which is due to people who were already in poverty being pushed further into poverty. Similarly, rural residents and female-headed households had a higher proportion of individuals that were pushed below the national poverty line compared to their counterparts. It is important to note that the proportion of people living below the national poverty line before deducting OOP health spending (37%) is seven percentage points higher than the World Bank’s estimate (30%). Also, pre-payment poverty rates for the regions were also estimated at higher ratios [24]. These disparities may be due to the difference in data sources. Nevertheless, these estimates show that about a third of the Ethiopian population is living below the national poverty line.

The results of this paper are not directly comparable with other studies due to differences in methodology, poverty lines, and/or data. Nevertheless, the results appear similar to those from some studies. A recent international study showed that impoverishment resulting from OOP health spending ranged between 1% point and 4% points [3]. An earlier study using the 2004 data showed that OOP health spending impoverished less than 1% of the Ethiopian population [17]. While that study used older data, the more recent (2010/11) HCES dataset shows that impoverishment from OOP health spending in Ethiopia is as high as 1.19% points. Other studies in Africa found a similar pattern of impoverishment from OOP health spending [28–32]. For example, in Ghana, J Akazili, JE-O Ataguba, EW Kanmiki, J Gyapong, O Sankoh, A Oduro and D McIntyre [31] report that between 1.6% points and 1.8% points of the Ghanaian population were impoverished by OOP health spending in 2005/06. In eSwatini, this was estimated to be between 1.0 and 1.6% of the Swazi population in 2009/10 [32]. B Kwesiga, CM Zikusooka and JE Ataguba [30] found that as high as 4% of the Ugandan population was impoverished by OOP health spending in 2009/10.

The impoverishing effect of OOP spending in Ethiopia suggests that policies such as the fee waiver and health insurance schemes [33] have not led to reductions in the incidence of impoverishment from paying OOP for health services. Fee waiver beneficiaries still report under-utilisation and other barriers to seeking health services [34]. Although introduced since 2003/04, the new fee-waiver system was not fully operational nationally in 2011 with only about 6% of people living below the poverty line screened [34]. Based on the 2011 Ethiopian population, these were only about 1.8 million people [27]. All things being equal, any individual from the remaining population living below the poverty line (22.9 million) would be pushed further into poverty as a result of OOP health spending, if health services are needed. Fee-waiver does not also necessarily provide the safety net it is supposed to. It was revealed that fee waiver beneficiaries pay for healthcare despite being entitled to free care due to perceived poor quality of free services, unavailability of drugs and diagnostic procedures, and to avoid the social stigma of being labelled the poorest [35]. Previous studies also show that fee-waiver and exemption systems are ineffective in providing financial protection to the poor [33, 36, 37]. It should also be noted that the fee-waiver system does not cover non-medical costs of care-seeking (e.g. transportation, food, and accommodation), and the opportunity cost of care-seeking. These costs can be substantial and can increase the burden of healthcare payments [38]. Thus, impoverishing healthcare payments may lead to forgoing needed healthcare services, which further aggravates health problems. Households may also resort to coping strategies such as borrowing, distress sale of assets, and reducing consumption of necessities [39]. Therefore, OOP health spending is a barrier to achieving not only the health-related SDGs but also the other goals such as ending poverty, hunger, and food insecurity by trapping people in the vicious cycle of poverty [4].

Similar to people below the poverty line who are pushed deeper into poverty due to OOP health spending, the burden faced by other populations – especially those near poor – should also be appreciated. Households who are not entitled to fee-waiver or covered by health insurance and are closer to the poverty line can easily fall into poverty if they incur OOP healthcare costs. Therefore, they would also be either ignoring healthcare needs or using similar coping strategies to reduce healthcare payments and thereby avoid poverty. This study showed that the risk of impoverishment is higher for rural residents than for urban residents. This can be explained by the interrelated factors of the higher burden of disease in rural areas coupled with inadequate means for paying for healthcare services due to subsistence living [40–42]. Sub-populations that are vulnerable to impoverishment from OOP health spending include households with many dependents (either large household size) and/or households with under-five children. Under-five children may need more healthcare services, which increase OOP health spending [43]. Among household characteristics that are associated with a lower probability of impoverishment are living in households headed by a male and/or educated persons. Other studies from China, India, Nigeria, and Vietnam [44–47] reported similar results. This is an indication of gender-based differences in the burden and impact of OOP health spending to the disadvantage of women. This also highlights the importance of the social determinants of health in influencing healthcare need and thereby OOP health spending and impoverishment. Based on this study, age and having older people in a household were not related to impoverishment. However, K Koch, C Pedraza and A Schmid [46] and S Ahmed, S Szabo and K Nilsen [47] found that having older adults and a household headed by older persons increase the likelihood of impoverishment from OOP health spending. It remains unclear why these variables were not significant in the Ethiopian case.

Although the Ethiopian health insurance reforms were at their early stages when the data used for this study were collected, a study that evaluated the CBHI pilot programmes provides some insights on the affordability of the schemes. The evaluation showed that although premiums were subsidised, especially for the poorest of the poor, 39% of non-CBHI members indicated the unaffordability of premium as a barrier to enrolment. Premiums were also unaffordable for about 16% of CBHI members [34]. In fact, between 2012 and 2013, more than a quarter (26%) of households in the pilot districts, mainly those in the lowest income quintile, dropped out of CBHI schemes because they could not afford to pay the monthly premiums [48]. Thus, the estimated impoverishing impact of OOP health spending reported in this paper may be an underestimation [36].

Although this study used the 2011 data, the latest HCES data that were available at the time of the study, the results reported in this paper are very likely not to have changed significantly as OOP payments remained high in 2016; accounting for 37% of current health expenditure [49]. Based on the results of this paper, there is a need for the government of Ethiopia to aim to reduce the share of OOP health spending in overall health financing in the country. This reduction is achievable through a well-functioning pre-payment system for health services. If there is no reduction in OOP health spending, all things being equal, more than 1.2 million people will be pushed into poverty in 2020 based on poverty headcount estimates of this study and population projections for 2020 [27]. Thus, prepayment systems need to be expanded urgently. Lessons from the CBHI pilot and other health financing reforms could be used to design prepayment systems that guarantee access to affordable and effective health services for all in Ethiopia. One challenge for providing adequate financial protection in Ethiopia is the large share (> 80%) of the population engaged in either rural agrarian activities or the informal sector [41, 50]. This notwithstanding, Ethiopia can consolidate on the already declining share of OOP health spending in current health financing and the SHI framework to ensure that financial protection is realised, especially for low-income households and those that are unable to afford health services through an effective cross-subsidisation mechanism. The strength of this paper is the use of a nationally representative and the latest available HCES data. The study also presents a comparative analysis of poverty levels using both international and national poverty lines. There are also a few limitations. OOP data may have been under-reported since health expenditure was aggregated at the household level and due to recall bias. The survey also excluded those who did not seek needed health care due to unaffordability. Thus, impoverishment resulting from OOP health spending may have been under-estimated.

## Conclusion

This study shows that a significant number of the population in Ethiopia is impoverished by paying OOP for health services. Generally, OOP health spending impoverishes the population by either pushing the non-poor below the poverty line or deepening poverty levels of the poor. Recent reforms such as the fee waiver and CBHI scheme seem to have contributed minimally to increasing financial protection in the country. Thus, households who were already below the poverty line and who needed health care have to bear further impoverishment or forgo healthcare services. The Ethiopian government needs to do more to address the challenges, especially for the poor and those who are unable to afford health services. Government actions may include, among other things, scaling up the uptake of health insurance and ensuring universal health coverage for all in Ethiopia. Where possible, the large informal sector may suggest that an alternative non-contributory prepayment system may be significant in providing financial protection for all in Ethiopia.

## Supplementary information

**Additional file 1:Table 3.** The impoverishing effect of OOP health spending by region in Ethiopia, 2010/11. Table 4: The impoverishing effect of OOP health spending by residential areas and gender in Ethiopia 2010/11.

## Data Availability

The dataset analysed for this study can be obtained from the Ethiopian Central Statistical Agency.

## Abbreviations

CBHI: Community-based health insurance
EA: Enumeration areas
FDRE: Federal Democratic Republic of Ethiopia
HCES: Household consumption and expenditure survey
OOP: Out-of-pocket
SDGs: Sustainable development goals
SSA: Sub-Saharan Africa
WHO: World Health Organization

## Footnotes

1 The Global Health Expenditure dataset estimates OOP payments at 47% of current health expenditure in 2011. https://apps.who.int/nha/database/ViewData/Indicators/en
