# Supplementary material for: Assessing medical impoverishment and associated factors in health care in Ethiopia
Source: BMC Int Health Hum Rights. 2020 Mar 30;20:7. doi: 10.1186/s12914-020-00227-x (PMC7106681; doi:10.1186/s12914-020-00227-x)
Supplement: Supplementary file 1 — Additional file 1:Table 3. The impoverishing effect of OOP health spending by region in Ethiopia, 2010/11. Table 4: The impoverishing effect of OOP health spending by residential areas and gender in Ethiopia 2010/11. [file 12914_2020_227_MOESM1_ESM.docx]

Table 3: The impoverishing effect of OOP health spending by region in Ethiopia, 2010/11

|  |  | Tigray | Afar | Amhara | Oromia | Somali | Benishangul-Gumuz | SNNP | Gambella | Harari | Addis Ababa | Dire Dawa |
| --- | --- | --- | --- | --- | --- | --- | --- | --- | --- | --- | --- | --- |
| Poverty headcount | Pre | 37.30% | 40.93% | 48.14% | 43.48% | 43.74% | 42.33% | 54.92% | 37.46% | 12.31% | 11.49% | 24.85% |
|  | Post | 37.69% | 41.51% | 49.39% | 44.58% | 45.10% | 43.45% | 56.59% | 38.54% | 14.66% | 11.84% | 25.66% |
|  | Absolute difference | 0.39% | 0.58% | 1.25% | 1.10% | 1.36% | 1.12% | 1.66% | 1.08% | 2.35% | 0.35% | 0.81% |
|  | Relative difference | 1.06% | 1.42% | 2.59% | 2.53% | 3.11% | 2.65% | 3.03% | 2.89% | 19.06% | 3.04% | 3.26% |
| Poverty gap | Pre | 370.19 | 393.26 | 546.99 | 476.91 | 422.71 | 452.98 | 652.60 | 372.98 | 74.95 | 99.21 | 138.44 |
|  | Post | 385.84 | 415.18 | 565.92 | 502.53 | 437.01 | 474.19 | 679.62 | 387.71 | 83.14 | 108.62 | 144.75 |
|  | Absolute difference | 15.65 | 21.92 | 18.93 | 25.63 | 14.30 | 21.21 | 27.02 | 14.73 | 8.19 | 9.41 | 6.31 |
|  | Relative difference | 0.04 | 0.06 | 0.03 | 0.05 | 0.03 | 0.05 | 0.04 | 0.04 | 0.11 | 0.09 | 0.05 |
| Normalised gap | Pre | 9.79% | 10.40% | 14.47% | 12.61% | 11.18% | 11.98% | 17.26% | 9.86% | 1.98% | 2.62% | 3.66% |
|  | Post | 10.20% | 10.98% | 14.97% | 13.29% | 11.56% | 12.54% | 17.97% | 10.25% | 2.20% | 2.87% | 3.83% |
|  | Absolute difference | 0.41% | 0.58% | 0.50% | 0.68% | 0.38% | 0.56% | 0.71% | 0.39% | 0.22% | 0.25% | 0.17% |
|  | Relative difference | 4.23% | 5.57% | 3.46% | 5.37% | 3.38% | 4.68% | 4.14% | 3.95% | 10.92% | 9.48% | 4.56% |
| Normalised mean positive gap | Pre | 26.25% | 25.41% | 30.05% | 29.01% | 25.56% | 28.30% | 31.43% | 26.34% | 16.10% | 22.84% | 14.73% |
|  | Post | 27.07% | 26.45% | 30.30% | 29.82% | 25.63% | 28.86% | 31.77% | 26.61% | 15.00% | 24.27% | 14.92% |
|  | Absolute difference | 0.82% | 1.04% | 0.26% | 0.80% | 0.07% | 0.56% | 0.34% | 0.27% | -1.10% | 1.43% | 0.19% |
|  | Relative difference | 3.14% | 4.10% | 0.85% | 2.77% | 0.26% | 1.98% | 1.08% | 1.03% | -6.84% | 6.25% | 1.26% |

Table 4: The impoverishing effect of OOP health spending by residential areas and gender in Ethiopia 2010/11

|  |  | Urban | Rural | Male | Female |
| --- | --- | --- | --- | --- | --- |
| Poverty headcount | Pre | 14.99% | 51.59% | 46.54% | 38.70% |
|  | Post | 15.59% | 52.90% | 47.70% | 40.01% |
|  | absolute difference | 0.60% | 1.31% | 1.16% | 1.31% |
|  | relative difference | 3.98% | 2.54% | 2.50% | 3.39% |
| Poverty gap | Pre | 127.35 | 585.45 | 521.14 | 429.58 |
|  | Post | 135.43 | 611.35 | 544.52 | 449.55 |
|  | absolute difference | 8.08 | 25.90 | 23.38 | 19.98 |
|  | relative difference | 0.06 | 0.04 | 0.04 | 0.05 |
| Normalised gap | Pre | 3.37% | 15.48% | 13.78% | 11.36% |
|  | Post | 3.58% | 16.17% | 14.40% | 11.89% |
|  | absolute difference | 0.21% | 0.69% | 0.62% | 0.53% |
|  | relative difference | 6.34% | 4.42% | 4.49% | 4.65% |
| Normalised mean positive gap | Pre | 22.47% | 30.01% | 29.62% | 29.36% |
|  | Post | 22.98% | 30.56% | 30.19% | 29.71% |
|  | absolute difference | 0.51% | 0.55% | 0.57% | 0.36% |
|  | relative difference | 2.27% | 1.84% | 1.94% | 1.22% |
